# Cations and Phenolic Compounds Concentrations in Fruits of Fig Plants Exposed to Moderate Levels of Salinity

**DOI:** 10.3390/antiox10121865

**Published:** 2021-11-24

**Authors:** Alessandra Francini, Mirko Sodini, Giulia Vicario, Andrea Raffaelli, Riccardo Gucci, Giovanni Caruso, Luca Sebastiani

**Affiliations:** 1 BioLabs, Institute of Life Science, Scuola Superiore Sant’Anna, Piazza Martiri della Libertà 33, 56127 Pisa, Italy; a.francini@santannapisa.it (A.F.); sodinimirko@hotmail.it (M.S.); g.vicario@santannapisa.it (G.V.); andrea1.raffaelli@santannapisa.it (A.R.); 2 Department of Agriculture, Food and Environment, University of Pisa, Via del Borghetto 80, 56124 Pisa, Italy; riccardo.gucci@unipi.it (R.G.); giovanni.caruso@unipi.it (G.C.)

**Keywords:** antioxidants, epicatechin, *Ficus carica*, phenols, micronutrients, salt stress

## Abstract

Fig trees are often grown in areas affected by salinity problems. We investigated changes in the concentrations of 15 phenolic compounds and mineral elements (Mg, Ca, K, Zn, Cu, Mn, Mo, Fe, Na) in fruits of fig plants (*Ficus carica* L. cv. Dottato) subjected to irrigation with saline water (100 mM of NaCl) for 28 days. We used UHPLC-MS/MS techniques to determine chlorogenic acid, tiliroside, catechin, epicatechin (ECTC), *p*-coumaric acid, trans-ferulic acid, phloridzin, phloretine, quercetagetin 7-*O*-glucoside, rutin, quercetin 3-*O*-glucoside, kaempferol 3-*O*-rutinoside, kaempferol 7-*O*-glucoside, kaempferol 3-*O*-glucoside, and quercetin. There was a steep gradient of Na^+^ concentrations between the root and the canopy of salinized plants, but leaf Na^+^ was similar in control and salt-treated plants. Quercetin, ECTC, and chlorogenic acid were the most abundant phenolic compounds in fig fruits. Salinity increased total phenols by 5.6%, but this increase was significant only for ECTC. Salt stress significantly increased Zn and Mg concentration in the fruit. Leaf levels of K, Mg, Ca, and Mn were similar in control and salinized plants. Moderate salt stress appears to improve fig fruit quality because of its positive effect on nutrients and antioxidant compounds such as epicatechin.

## 1. Introduction

Fig trees have been grown for their fruits since ancient times in Southwest Asia and the Mediterranean basin [1]. In these regions, salinity and long periods of drought are often limiting factors for the survival and productivity of perennial crops. It is also likely that water shortage and salinity will further threaten plant performance in the near future as a result of climate change [2,3]. 

Stress conditions determine a number of physiological and biochemical responses in plants. One of the main consequences of drought or salinity is oxidative stress that triggers a cascade of damaging events in cells [4,5]. The role of antioxidant compounds synthesized and accumulated in plant organs becomes crucial to cope with stress. Pigments, non-enzymatic antioxidants, and antioxidative enzymes are all active against oxidative stress [6] as well as salinity [7]. Salinity affects the physiology, metabolism, and gene expression of fig trees [8,9,10]. In general, saline stress decreases plant water potential, photosynthetic rate, stomatal conductance, growth, and alters biomass partitioning and ion distribution within the plant [8,11,12,13]. The role of proline as an osmoprotectant during salt stress [11,14] is not evident in all cultivars [15]. 

Fig leaves and fruits are particularly rich in phenolic compounds with antioxidant activities [16,17,18]. Similarly to other species, pigments and antioxidant enzymes play a key role to counteract the effect of water stress in fig trees [19,20], but the effect is genotype dependent. In a study where four Iranian cultivars were compared, ‘Deyme Ahvaz’ (Deym), ‘Sabz Estahban’ (Sabz), ‘Siah’, and ‘Shahanjir’ (Shah), it was reported that Deym had higher pigment content and activities of antioxidant enzymes than other cultivars. Moreover, water stress significantly increased α-tocopherol concentration, but decreased ascorbic acid content [21].

Fruit characteristics and composition of fig plants grown under saline conditions are poorly defined. A recent article reported that salinity increased sucrose and sorbitol concentrations in fig fruits, but not glucose or fructose [9]. The expression levels of the main sugar metabolism and transport genes were studied in fig tree under salt stress [9,22]. In general, transcript levels of genes involved in the transport of soluble sugars in ripe fig fruits were affected by salinity [9]. Alkaline-neutral and acid invertases transcripts were upregulated in salinized plants, as well as the transcription of sucrose-synthase and sorbitol dehydrogenase encoding genes [9]. Other molecular studies showed that the introduction, via Agrobacterium-mediated transformation into the fig cultivar Black Mission, of the gene *AgNHX1* (codifying a vacuolar Na/H antiporter in the halophytic species *Atriplex gmelini*) conferred high tolerance to salinity [14]. Moreover, a RNA seq transcriptome analysis at 24 and 48 days on cultivar Dottato exposed to salt stress showed the activation of several genes related to the adaptation to salt stress and in particular of those involved in the production of leaf secondary metabolites [10].

Beside its nutritional value, the fig fruit has many beneficial effects on human health including antioxidant, anti-inflammatory [23], and anti-diabetic properties [24], as well as anti-bacterial activities [25]. In particular, the fig fruit is rich in sugars, amino acids, minerals, vitamins, and secondary metabolites. As for nutrients, the fruit is low in sodium (Na) and high in potassium (K), calcium (Ca), magnesium (Mg), iron (Fe), and zinc (Zn) concentrations [26,27,28]. Different classes of antioxidant molecules, such as phenols, carotenoids, and tocopherols are present [27]. Among phenolic compounds, phenolic acids and flavonoids (flavanols, flavonols, and anthocyanidins) were identified [16,17,29]. Since their concentrations vary according to genetic and environmental factors [18,30], irrigation with saline water may induce a mild stress and the accumulation of health-promoting phytochemicals in plant organs [31] or improve fruit quality by increasing dry matter, soluble solids, and carbohydrates, as it happens in other crops [32,33].

The effects of saline water on fruit mineral nutrients and secondary metabolite composition in fig fruits has not been investigated. The aim of our work was to determine the effect of moderate salinization (100 mM NaCl for four weeks) on mineral elements and phenolic compounds of fig fruits (cv. Dottato). In particular, we specifically addressed whether short periods of irrigation with saline water could improve fruit nutraceutical quality by stimulating the accumulation of antioxidants and nutrients.

## 2. Materials and Methods

### 2.1. Plant Material and Salt Treatment

Three-year-old fig plants (*Ficus carica* L., cultivar Dottato) from self-rooted cuttings were grown in 9.5 L pots (0.3 m diameter) filled with a mixture of 6.4% clay, 8.6% silt, and 85% sand. Plants were acclimated for three weeks in a growth chamber (Phytotron, Monti & C., Pistoia, Italy) at 400–500 µmol m^−2^s^−1^ photosynthetic photon flux density, 26/20 °C day/night temperature, 55/75% day/night relative humidity, and a 16/8 h photoperiod. Plants were randomly arranged within the growth chamber and their height, the number of fully-expanded leaves, and the number of fruits were measured. Two groups of six plants, homogeneous in size, were then assigned to treatments. Prior to the beginning of salinization, average plant height was 1.04 ± 0.08 m and each plant had 13 ± 4 leaves and 4 ± 2 fruits. Control plants were irrigated to soil capacity with tap water, whereas salinized ones received water added with 100 mM of NaCl (purity > 99.8%) twice a week for four weeks. In order to leach salinity in the pot, irrigation water was supplied in excess.

At the end of the salinization period, plants were partitioned into fruits, leaves, roots, and stems. Stem portions were excised at 50 mm from the apex (high stem) and at 50 mm from the collar (low stem). The fruits were further divided into pulp and peel. Half of the plant material was frozen in liquid nitrogen and then stored at −80 °C, and the remaining half was weighed and then dried in an oven (70 °C) to constant weight. The latter samples were analyzed for cations concentration, water content, and biomass dry weight.

### 2.2. Cation Concentration

Macro- and micro-element (Mg, Ca, K, Zn, Cu, Mn, Mo, Fe, and Na) concentrations were determined on completely dry samples. A total of 0.2 g of plant material were digested in 5 mL of 65% HNO_3_ and 10 mL of 70% HClO_4_. The resulting solution was filtered, diluted with Milli-Q H_2_O, and then analyzed. 

A Microwave Plasma-Atomic Emission Spectrometer (4210 MP-AES, Agilent Technologies, Santa Clara, CA, USA) was used for the element quantification and *Daucus carota* (L.) leaf was the analytical certified standard (WEPAL IPE, Wageningen University, Wageningen, The Netherlands). Multi-element calibration standards were prepared for macro- and micro-element determinations. All standards were prepared in 5% HNO_3_ (*v*/*v*) medium and diluted with Milli-Q H_2_O.

### 2.3. Proline Concentration 

The proline concentration was determined according to the method of Bates et al. [34] with minor modifications. Half a gram of fresh leaf tissue stored at −80 °C was homogenized with 5 mL of 3% aqueous sulfosalicylic acid (*v*/*v*). The homogenized solution was filtered, and 1 mL was added to an equal volume of glacial acetic acid and ninhydrin. The mixture was incubated at 100 °C for 1 h, and the reaction terminated in an ice bath before extraction with 2 mL of toluene. The chromophore was removed from the toluene phase, and the absorbance read at 520 nm using a spectrophotometer (Tecan Infinite^®^ 200 PRO, Männedorf, Switzerland). The proline concentration was determined in the range of 0.1–10 µmol using a standard curve.

### 2.4. Fruit Phenolic Compounds 

Phenolic compounds were extracted pooling three fruits per plant. Six plants per treatments (*n* = 6) were used. The samples were prepared according to the method reviewed by Arvaniti et al. [27] with the following modifications. An average of 2 g of fresh whole fruit was extracted in 10 mL of a 80:20 (*v*/*v*) methanol:water solution, and shaken in the dark for 2 h. The extracts were then centrifuged at 2000× *g* for 20 min in a swing bucket Rotor (benchtop Eppendorf 5804, Milan, Italy). Afterwards, the supernatant was collected and filtered through a Whatman (Puradisc, 0.45 µm) cartridge. The extract obtained was diluted 1:20 with MilliQ water before UHPLC-MS/MS analyses.

The extracts were subjected to targeted quantitative analyses of selected known polyphenols by UHPLC-MS/MS using a Sciex 5500 QTrap+ mass spectrometer (AB Sciex LLC, Framingham, MA, USA), equipped with a Turbo V ion-spray source and coupled to an ExionLC AC System custom made by Shimadzu (Shimadzu Corporation, Kyoto, Japan) which includes an ExionLC Controller, ExionLC Degasser, ExionLC Tray, 2 ExionLC AC Pumps, and ExionLC AC Autosampler.

Chromatographic separation was performed using a Phenomenex Kinetex EVO 2 × 100 mm, 5 µm column (Phenomenex, Torrance, CA, USA). The elution was carried out in gradient mode using acetonitrile containing 0.1% formic acid (solvent A) and water containing 0.1% formic acid (solvent B). The gradient elution was programmed as follows: 0.0 min, A 5%; 0.0–10.0 min, A 5–95%; 10.0–12.0 min, A 95% and followed by 4 min equilibration time (A 5%). Other chromatographic conditions were flow rate 300 µL min^−1^, injection volume 20 µL, and column oven temperature 40 °C.

MS/MS experiments were performed in Electrospray negative ion mode using nitrogen as collision gas, with the operation source parameters: source type, Turbospray; nebulizer gas (GS1) 70 (arbitrary units); turbo gas (GS2) 50 (arbitrary units); curtain gas (CUR) 10 (arbitrary units); temperature (TEM) 500 °C; Ionspray Voltage (IS) −4500 V, entrance potential (EP) 10 V. Compound parameters, declustering potential (DP), collision energy (CE), and collision cell exit potential (CXP) were adjusted for the specific Selected Reaction Monitoring (SRM) transition for any component. SRM transitions and the corresponding compound parameters are reported in the Table 1.

Data were normalized according to matrix effect and recovery percentage. Matrix effect was calculated as the peak area of the sample spiked after extraction/peak area of the standard, while recovery was calculated as the peak area of the sample spiked before extraction/peak area of the sample spiked after extraction. Each sample was replicated three times. 

Qualitative confirmation was obtained taking advantage of one of the features of a QTrap instrument. Information Dependent Acquisition (IDA) was programmed so that the SRM transitions reported in Table 1 were used as a survey scan, switching the third quadrupole to act as a Linear Ion Trap (LIT), performing an Enhanced Product Ions (EPI) scan, affording the complete MS-MS product ions spectrum (MRM >> Enhanced Product experiment). A comparison with a custom-built MS-MS product Ions spectra library allowed the qualitative confirmation. 

### 2.5. Statistical Analysis

All data were expressed as means ± standard deviations (SD). Differences between means in control and NaCl-treated plants were assessed by *t*-test, with differences being considered significant at *p* ≤ 0.05. Correlations among parameters were analyzed using Pearson’s correlations (*p* < 0.05 and *p* < 0.01). Box-and-whisker plots were also used [35].

## 3. Results 

After four weeks of saline treatment at 100 mM, shoot length increment and fruit fresh weight were not significantly different from those of controls (Table 2). The leaf proline content of both treatments was also similar (Table 2).

**Figure 1:**
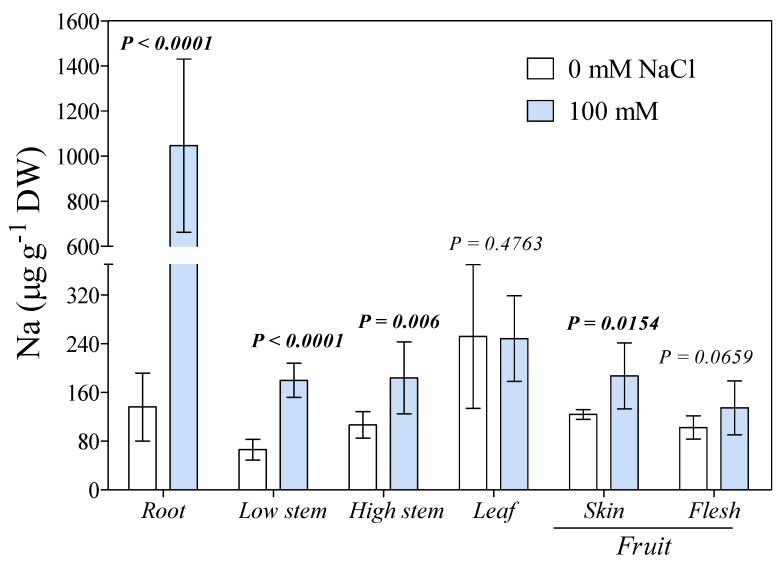
Sodium concentration in leaf, stem, root, and fruits (skin and flesh) of fig plants treated with 0 or 100 mM of NaCl for 28 days. Statistical significance and *p*-values were determined within the same organ comparing the two treatments (*n =* 6) by *t*-test.

**Figure 2:**
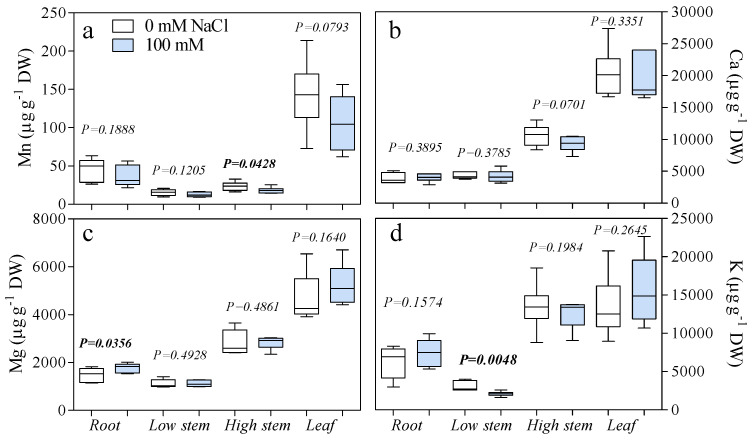
Concentrations of (**a**) Mn, (**b**) Ca, (**c**) Mg, and (**d**) K (µg g^−1^ of dry weight), in leaf, stem (low and high), and root of fig plants treated with 0 and 100 mM of NaCl for 28 days. Statistical significance and *p*-values were determined within the same organ comparing the two treatments (*n* = 6) by *t*-test. Box-and-whisker plot showing mean, minimum-maximum range, and 25–75 percentile range.

Sodium was uptaken by the roots and translocated into the canopy. The root Na concentrations of salinized plants was over 1000 µg g^−1^ DW, a value 7.7-fold higher than in the controls (Figure 1). Stem Na^+^ concentrations (both lower and upper parts) of salt-treated plants were also significantly higher than those of control ones, but the gap between treatments was less than in the roots. The stem Na^+^ concentration of plants exposed to 100 mM NaCl was 196 and 180 µg g^−1^ DW in the lower (next to the crown) and upper part (next to the apex), respectively (Figure 1). Interestingly, leaf Na^+^ concentrations were similar for both treatments. As for the fruit, Na^+^ concentrations were significantly higher in the skin, but not in the flesh of salinized plants. Skin Na^+^ concentration was 51% higher than in fruits from control plants. The Mn, Mg, K, and Ca concentrations in leaves, stem, and roots were not significantly different between the two treatments, except for Mg in roots, Mn in the upper portion of the stem, and K in the lower part (Figure 2).

**Figure 3:**
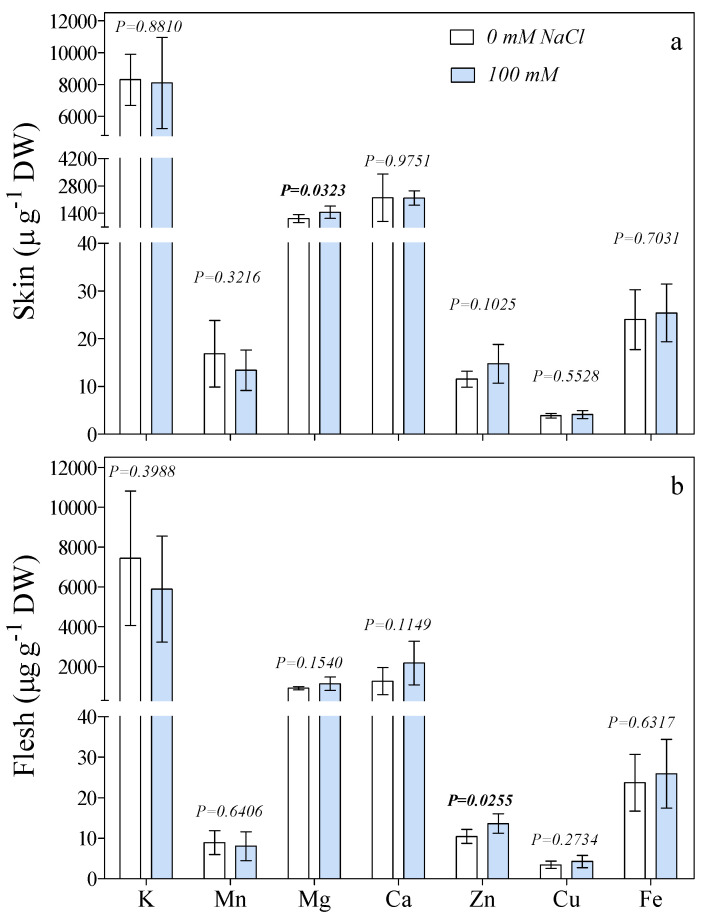
Concentrations of K, Mn, Mg, Ca, Zn, Cu, and Fe in the skin (**a**) or flesh (**b**) of fig fruits treated with 0 or 100 mM NaCl for 28 days. Statistical significance between treatments was determined within the same organ and treatments (*n* = 6) were compared by *t*-test.

The concentrations of K, Mn, Mg, Ca, Zn, Cu, and Fe in the fruit (skin and flesh) of plants exposed to 0 mM and 100 mM NaCl are reported in Figure 3. The elemental composition of the skin and the flesh was similar. K, Ca, and Mg were the most abundant cations. Their concentrations were similar for both treatments, except for Mg, which was higher in the skin of 100 mM NaCl-treated fruits (Figure 3). The Mg concentration was significantly increased (+28%) by the salt treatment (from 1132 to 1453 µg g^−1^ DW in 0 mM and 100 mM NaCl, respectively). As for micronutrients, only Zn showed a significantly higher concentration in NaCl-treated fruits than in control ones, whereas Mn, Cu, and Fe had similar values for both treatments. Zinc reached 13 µg g^−1^ DW in the flesh of the 100 mM NaCl fruit, a +30% increase in comparison with the control (10 µg g^−1^ DW). Zinc concentration was also higher in the skin of salinized fruits, but this difference was not significant (Figure 3).

**Figure 4:**
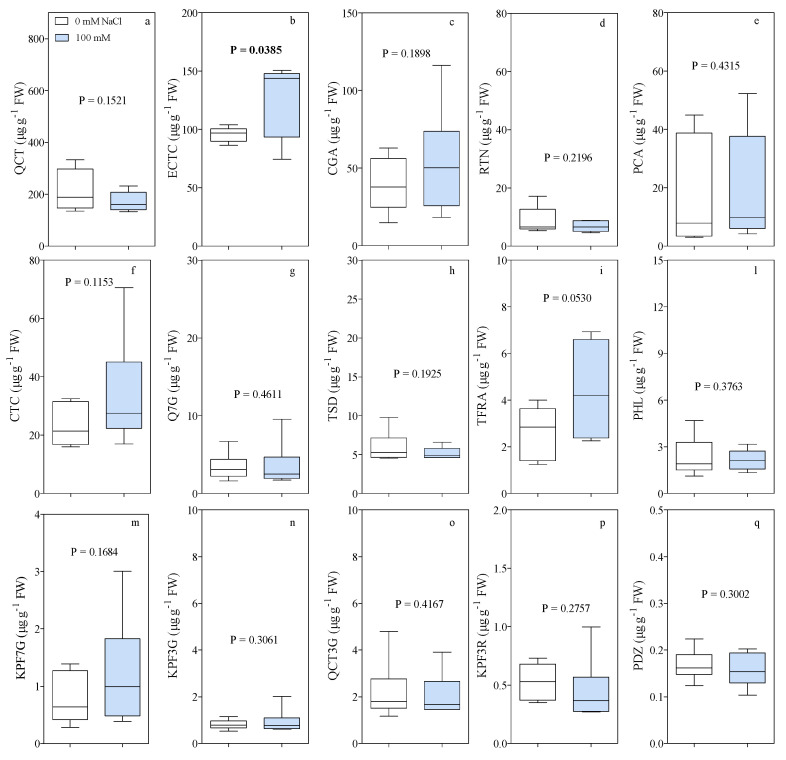
Mean values and standard deviation for concentration (µg g^−1^ FW) of (**a**) Quercetin (QCT), (**b**) (−)-Epicatechin (ECTC), (**c**) Chlorogenic Acid (CGA), (**d**) Rutin (RTN), (**e**) *p*-Coumaric Acid (PCA), (**f**) (+)-Catechin (CTC), (**g**) Quercetagetin 7-*O*-Glucoside (Q7G), (**h**) Tiliroside (TSD), (**i**) Trans-ferulic Acid (TFRA), (**l**) Phloretin (PHL), (**m**) Kaempferol 7-*O*-glucoside (KPF7G), (**n**) Kaempferol 3-*O*-glucoside (KPF3G), (**o**) Quercetin 3-*O*-Glucoside (QCT3G), (**p**) Kaempferol 3-*O*-rutinoside (KPF3R), and (**q**) Phloridzin (PDZ), determined by the UHPLC-MS/MS analysis of fresh fruit material. Data (*n* = 6) were analyzed by *t*-test and probability level for significance is reported. Box-and-whisker plot showing mean, minimum-maximum range, and 25–75 percentile range.

A panel of 15 polyphenolic compounds, one cinnamate ester (chlorogenic acid), one oxyflavone (tiliroside), two flavanols (catechin and epicatechin), two hydroxy-cinnamic acids (*p*-coumaric and trans-ferulic acid), two dihydrochalcones (phloridzin and phloretine), and seven flavonols (quercetagetin 7-*O*-glucoside, rutin, quercetin 3-*O*-glucoside, kaempferol 3-*O*-rutinoside, kaempferol 7-*O*-glucoside, kaempferol 3-*O*-glucoside and quercetin), was selected for the antioxidative activity of these molecules and identified in fig fruits (Figure 4). A single SRM transition was used for the UHPLC-MS/MS analysis and to quantify the different concentrations.

**Figure 5:**
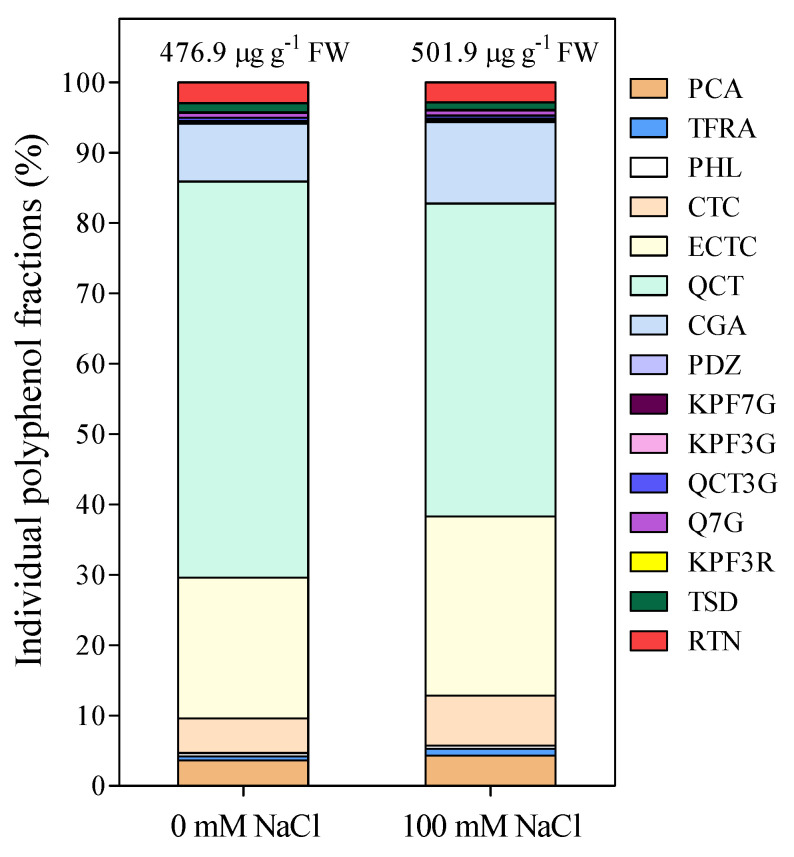
The relative proportion (%) of the 15 polyphenolic compounds analyzed in fig fruits of control (0 mM) and 100 mM NaCl-treated plants. Values were calculated as percentage of means (*n* = 6).

Quercetin, ECTC, and CGA were the most abundant phenolic compounds in fig fruits (Figure 4). Quercetin reached the highest concentration (212 and 273 µg g^−1^ FW for 0 mM and 100 mM-treated plants, respectively; Figure 4a). The lowest concentrations were measured for phloridzin (PDZ) with average values of 0.16 and 0.17 µg g^−1^ FW for the 0- and 100-mM treatment, respectively (Figure 4q). As expected, the fruit concentrations of the 15 polyphenols were quite variable. A significant increase induced by salinity was measured only for ECTC, while trans-ferulic acid (TFRA) was at the limit of significance (*p* = 0.053). Moreover, there was a shift in the total concentration (from 476.9 to 501.9 µg g^−1^ FW in 0 mM and 100 mM treated plants, respectively) and a change in the relative percentage of the individual phenolic compounds (Figure 5). In particular, some polyphenols such as PCA (3.6 vs. 4.3%), TFRA (0.6 vs. 1%), CTC (4.9 vs. 7.1%), ECTC (20 vs. 25.5), CGA (8.2 vs. 11.6), and KPF7G (0.2 vs. 0.3) increased their relative proportion in the fruit, while the proportion of others, such as QCT (56.3 vs. 44.5), QCT3G (0.5 vs. 0.4), TSD (1.2 vs. 1), and RTN (3 vs. 2.8), decreased (Figure 5). 

**Figure 6:**
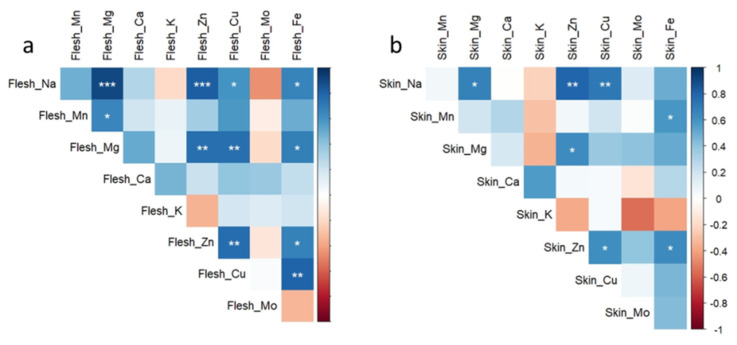
Correlation matrix based on Pearson’s correlation coefficients among flesh (**a**) and skin (**b**) mineral elements (Na, Mn, Mg, Ca, K, Zn, Cu, Mo, and Fe). Color intensity is proportional to Pearson’s coefficients. Positive correlations are in blue, and negative correlations in red. Significance of the Pearson’s coefficients is indicated by the asterisk: * = *p* ≤ 0.05, ** = *p* < 0.01, *** = *p* < 0.001.

The correlation matrix based on Pearson’s correlation coefficients among flesh (Figure 6a and Figure S1) and skin (Figure 6b and Figure S2) mineral elements showed a significant and positive correlation between flesh Na concentration and Mg (*r* = 0.907 and *p* ≤ 0.001), Zn (*r* = 0.825 and *p* ≤ 0.001), Cu (*r* = 0.597 and *p* ≤ 0.05), and Fe (*r* = 0.662 and *p* ≤ 0.05). These data are evidence that Na accumulation in the flesh is associated with a significant increase of Mg, Zn, Cu, and Fe concentrations. Na concentration in the skin was also positively correlated with Mg, (*r* = 0.674 and *p* ≤ 0.01) Zn (*r* = 0.798 and *p* ≤ 0.01), and Cu (*r* = 0.715 and *p* ≤ 0.01).

## 4. Discussion

Fig trees are moderately tolerant to salinity. Recent evidence showed that concentrations of 50 mM NaCl in the irrigation water could be tolerated for 7–8 weeks without symptoms of toxicity in the canopy of cv. Dottato. The safe range for plant growth and productivity of this cultivar is between 0 and 100 mM NaCl [8]. In fact, irrigation with 100 mM NaCl stopped growth after two weeks of treatment, but leaf turgor and chlorophyll content were maintained. Adjustments in water use efficiency, gas exchange parameters, and biomass accumulation allowed young plants to withstand salt stress at the root zone [8]. Although the physiology and gene expression of fig plants exposed to 100 mM NaCl for several weeks were clearly affected [9,10], preliminary evidence showed that short periods of salinity might be beneficial for the maintenance of turgor and accumulation of osmolytes [8].

In our current work, we confirmed that a concentration of 100 mM NaCl was well tolerated for four weeks since proline concentration, shoot, and fruit growth were similar to those of controls. Moreover, mineral elements in the control plant are in line with data measured in fig fruit [36] and other fig organs [37]. On the other hand, Na^+^ was massively accumulated in the root tissue and increased in all organs but not in the leaf. The steep gradient of Na concentration between the root and the canopy of salinized plants and the lack of any differences in Na^+^ concentration between controls and salt-treated leaves are evidence that fig plants probably exclude Na mainly at the root level, similarly to other moderately tolerant perennial crops [11,12]. However, the effectiveness of salt exclusion is dependent on the cultivar, stress duration, and plant age. Therefore, the high Na^+^ accumulation in the stem and leaf reported for younger plants exposed to salinity for a longer period [13] is still consistent with our results. The decreased concentration of K in the stem of the fig tree is also consistent with previous work [13,38,39] since Na competes with K for absorption due to electric charge and the atomic radius. Potassium is required by more than 50 enzymes and so K deficiency is one of the main problems caused by excessive Na^+^ [40]. It should also be noted that leaf levels of K, Mg, Ca, and Mn were maintained in fig plants salinized for four weeks in agreement with the good salt tolerance of this species. 

The main focus of our experiment was to investigate the impact of irrigation with saline water on fig fruit composition. Salt stress has been reported to increase the content in total phenols [7], hydroxybenzoic and hydroxycinnamic acids, and flavonoids [41]. Phenolic compounds play an important role as antioxidants in the scavenging of ROS species and decrease cell membrane peroxidation [42]. In our experiment, ECTC content in fig fruits was significantly higher in 100 mM NaCl-treated plants compared with the controls. Consistent with our results, a higher content of ECTC was measured in strawberry fruits of salt-stressed plants [43] and wheat sprouts (but not QCT, RTN, or KPF) when exposed to 50 mM NaCl [44]. Confirming the crucial role of flavonoids in mitigating salt stress damage, exogenous CTC applications were found effective in counteracting salt stress in *Capsicum annuum* L. seedlings [45]. In our salt-stressed plants, the concentration of QCT3G, CTC, ECTC, and CGA is enhanced in agreement with a general increase of phenolic compounds in response to salinity [46]. Catechins also appear to play a key role in response to other abiotic stresses like water deficit [47] and excess of heavy metals [48], as they are intermediates in anthocyanins biosynthesis.

Fruit Zn and Mg were increased by salinity, whereas other micronutrients were unaffected. The accumulation of Znmeasured in salt-treated fruits might have been a signal of the plant antioxidant response. Zinc has been widely reported to enhance the antioxidant capacity during salt stress [49,50]. Foliar applications of Zn alleviate the effect of salinity by reducing lipid peroxidation and increase the activity of antioxidant enzymes [51]. In addition, Zn is an essential component of superoxide dismutase Cu/Zn-SOD, which promotes the control of reactive oxygen species (ROS) [52]. Furthermore, Zn^2+^ is a cofactor of many enzymes promoting protein synthesis and metabolites production [50]. Therefore, the accumulation of Zn in the fruits of treated plants is consistent with the accumulation of phenolic compounds. 

There was a positive and significant correlation between Na concentration and those of Mg, Fe, and Cu. The mineral balance under salt stress is concentration dependent, but different species may respond differently [53]. Iron is a cofactor for chlorophyll synthesis, photosynthesis, and respiration. In plants, Cu has a similar role as Fe. Depending on its redox state, Cu can bind metabolites containing sulphur, oxygen, or imidazole nitrogen groups, so it participates in the transport of electrons in mitochondria and chloroplasts and in the remodeling of the cell wall [54]. Fe and Cu are also cofactors of SOD enzymes (the Cu/Zn superoxide dismutase and the Fe superoxide dismutase) located in different organelles and with a crucial role in ROS detoxification [55]. 

Because mineral elements and polyphenols play a relevant role in human nutrition and health [56,57], our results provide useful insights for improving the quality of fig fruits. The biosynthesis of phenylpropanoids is, in fact, stimulated by abiotic stresses, including salinity. This plasticity to abiotic stresses could be strategically used to change the polyphenolic profile and increase nutrient concentrations. Salinity also induced increases in sorbitol and sucrose concentrations in fig fruits [9]. Irrigation with saline water has been shown to increase soluble solids, carbohydrates, and organoleptic properties in annual crops like tomato and melon and it is used in commercial production in areas where water quality is not high [58,59]. 

## 5. Conclusions

In conclusion, salinization with 100 mM NaCl for 28 days determined sodium accumulation in roots, stems, and fruits. Moreover, shoot length increment, fruit fresh weight, and leaf proline content of salinity stressed fig were not significantly different from those of controls. Our data also proved that moderate salinity enables the accumulation of Zn and Mg in the fruit with potential benefits on fig nutritional quality. A similar trend was observed for the dietary polyphenol epicatechin that has been proved to have cardiovascular beneficial effects by epidemiological and clinical studies. Overall, mild salinity emerges as a positive stress for fig fruits’ nutritional and nutraceutical quality.

## Figures and Tables

**Table 1:** SRM transitions and relative compound parameters for targeted polyphenol compounds.

Name	Acronym	RT (min)	Q1	Q3	DP (V)	CE (eV)	CXP (V)
Catechin	CTC	2.33	289.0	244.9	−108	−22	−11
Chlorogenic Acid	CGA	2.44	353.0	191.0	−61	−24	−9
Epicatechin	ECTC	2.63	289.0	244.9	−108	−22	−11
Quercetagetin 7-*O*-Glucoside	Q7G	3.07	479.1	316.9	−152	−31	−14
*p*-Coumaric Acid	PCA	3.09	163.0	119.0	−65	−18	−11
Trans-Ferulic Acid	TFRA	3.28	193.0	134.0	−62	−20	−8
Rutin	RTN	3.30	609.2	299.9	−154	−48	−11
Quercetin 3-*O*-Glucoside	QCT3G	3.40	463.1	300.0	−154	−37	−5
Kaempferol 3-*O*-Rutinoside	KPF3R	3.53	593.2	284.9	−138	−40	−5
Kaempferol 3-*O*-Glucoside	KPF3G	3.62	447.1	284.1	−202	−39	−11
Kaempferol 7-*O*-Glucoside	KPF7G	3.66	447.1	284.9	−158	−38	−5
Phloridzin	PDZ	3.71	435.1	272.9	−135	−23	−5
Quercetin	QCT	4.30	301.0	150.9	−113	−38	−8
Tiliroside	TSD	4.41	593.2	284.9	−138	−40	−5
Phloretin	PHL	4.64	273.0	167.0	−103	−38	−11
Catechin	CTC	2.33	289.0	244.9	−108	−22	−11

**Table 2:** Shoot length increment, fruit fresh weight, and leaf proline content in fig plants treated with 0 and 100 mM of NaCl after 28 days of experiment. Values are means ± SD of six replicates. ns = not significant.

	Treatments (NaCl)		
	0 mM	100 mM	*t*-test
Shoot Length Increment (mm)	73 ± 15.4	56 ± 15.1	ns
Fruit Weight (g)	18.0 ± 5.88	16.1 ± 7.06	ns
Leaf Proline (mg g^−1^ FW)	0.28 ± 0.050	0.26 ± 0.030	ns

## Data Availability

Data is contained within the article.

## Supplementary Materials

The following are available online at https://www.mdpi.com/article/10.3390/antiox10121865/s1, Figure S1: Chart of a correlation matrix among flesh micro and macro elements. Figure S2: Chart of a correlation matrix among skin micro and macro elements.
