# Supplementary material for: Cations and Phenolic Compounds Concentrations in Fruits of Fig Plants Exposed to Moderate Levels of Salinity
Source: Antioxidants (Basel). 2021 Nov 24;10(12):1865. doi: 10.3390/antiox10121865 (PMC8698956; doi:10.3390/antiox10121865)
Supplement: Supplementary file 1 [file antioxidants-10-01865-s001.zip › antioxidants-1437524-supplementary.pdf]

Cations and phenolic compounds concentrations in fruits of fig plants exposed to moderate levels of salinity

Alessandra Francini[1#], Mirko Sodini[1#], Giulia Vicario[1], Andrea Raffaelli[1], Riccardo Gucci[2], Giovanni Caruso[2], and Luca Sebastiani[1*]

[1] BioLabs, Institute of Life Science, Scuola Superiore Sant'Anna, Piazza Martiri della Libertà 33, I-56127 Pisa, Italy (A.F.: a.francini@santannapisa.it; M.S.: sodinimirko@hotmail.it; G.V.: g.vicario@santannapisa.it; A.R.: andrea1.raffaelli@santannapisa.it; L.S.: l.sebastiani@santannapisa.it)

[2] Department of Agriculture, Food, and Environment, University of Pisa, Via del Borghetto 80, I-56124, Pisa, Italy (R.G.: riccardo.gucci@unipi.it; G.C.: giovanni.caruso@unipi.it)

* Correspondence: l.sebastiani@santannapisa.it (Luca Sebastiani)

[#] These authors contributed equally to this work

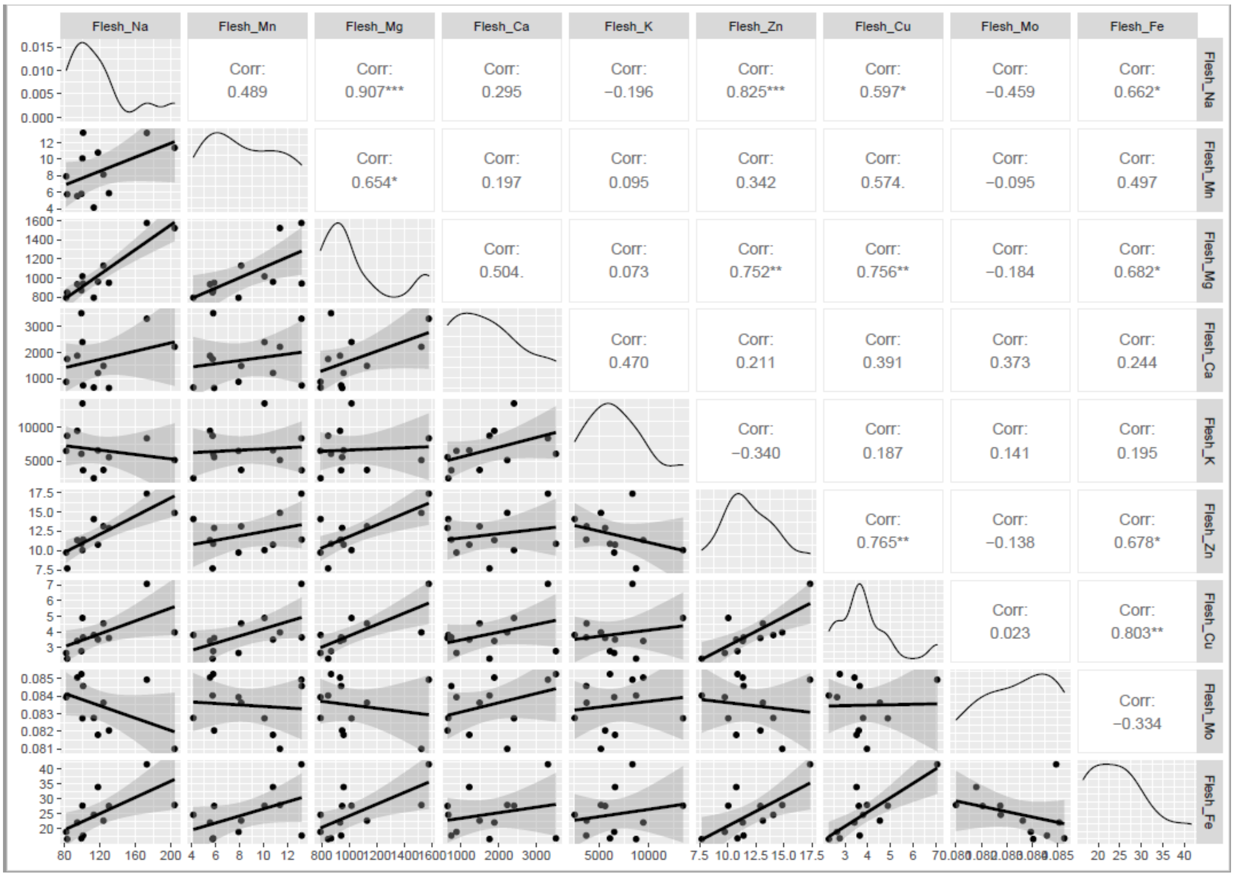

Figure S1. Chart of a correlation matrix among flesh micro- and macro-elements (ppm). The distribution of each variable is shown on the diagonal. Boxes below the diagonal report the bivariate scatter plots with fitted lines, those above the diagonal report correlation values.

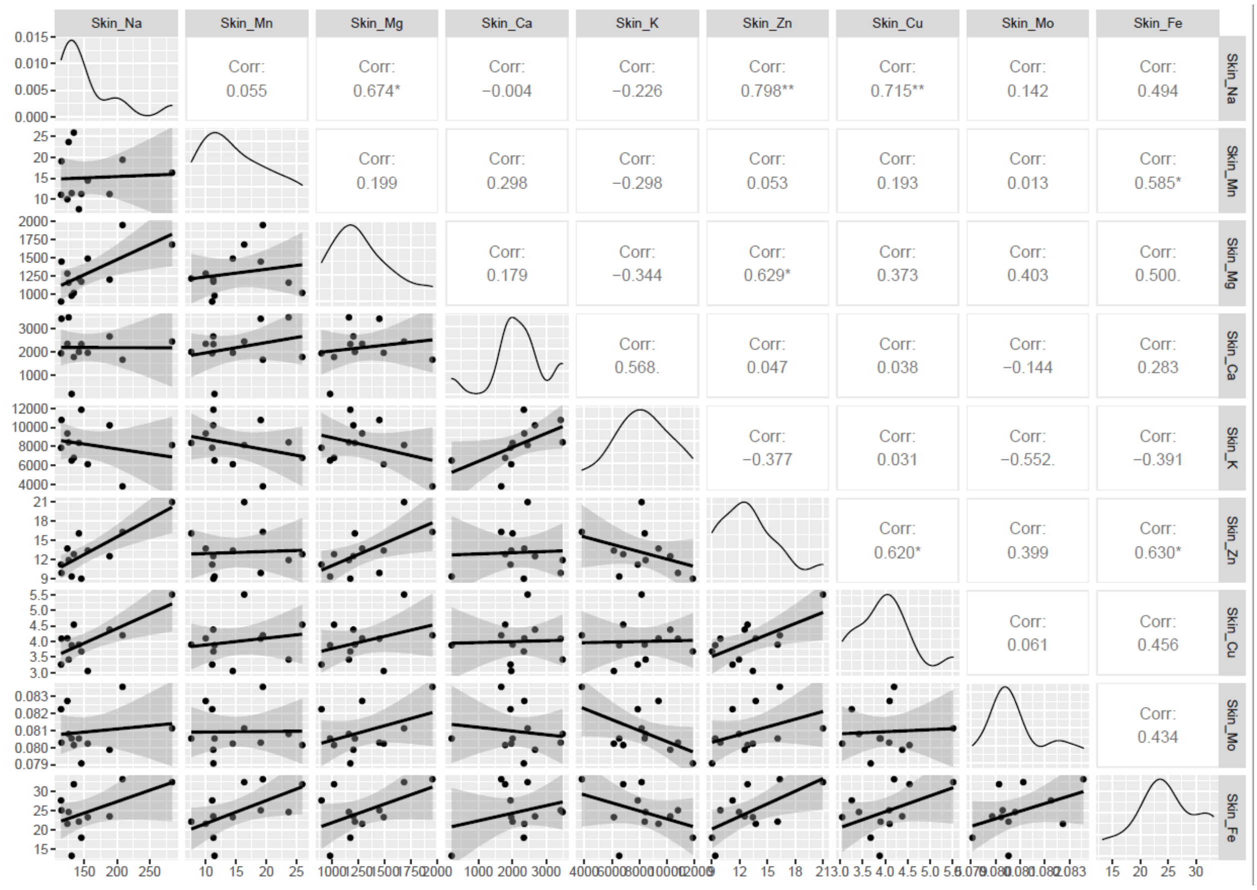

Figure S2. Chart of a correlation matrix among skin micro- and macro-elements (ppm). The distribution of each variable is shown on the diagonal. Boxes below the diagonal report the bivariate scatter plots with fitted lines, those above the diagonal report correlation values.
